# Complex Events Initiated by Individual Spikes in the Human Cerebral Cortex 

**DOI:** 10.1371/journal.pbio.0060222

**Published:** 2008-09-02

**Authors:** Gábor Molnár, Szabolcs Oláh, Gergely Komlósi, Miklós Füle, János Szabadics, Csaba Varga, Pál Barzó, Gábor Tamás

**Affiliations:** 1 Research Group for Cortical Microcircuits of the Hungarian Academy of Sciences, Department of Physiology, Anatomy and Neuroscience, University of Szeged, Szeged, Hungary; 2 Department of Neurosurgery, University of Szeged, Szeged, Hungary; Columbia University, United States of America

## Abstract

Synaptic interactions between neurons of the human cerebral cortex were not directly studied to date. We recorded the first dataset, to our knowledge, on the synaptic effect of identified human pyramidal cells on various types of postsynaptic neurons and reveal complex events triggered by individual action potentials in the human neocortical network. Brain slices were prepared from nonpathological samples of cortex that had to be removed for the surgical treatment of brain areas beneath association cortices of 58 patients aged 18 to 73 y. Simultaneous triple and quadruple whole-cell patch clamp recordings were performed testing mono- and polysynaptic potentials in target neurons following a single action potential fired by layer 2/3 pyramidal cells, and the temporal structure of events and underlying mechanisms were analyzed. In addition to monosynaptic postsynaptic potentials, individual action potentials in presynaptic pyramidal cells initiated long-lasting (37 ± 17 ms) sequences of events in the network lasting an order of magnitude longer than detected previously in other species. These event series were composed of specifically alternating glutamatergic and GABAergic postsynaptic potentials and required selective spike-to-spike coupling from pyramidal cells to GABAergic interneurons producing concomitant inhibitory as well as excitatory feed-forward action of GABA. Single action potentials of human neurons are sufficient to recruit Hebbian-like neuronal assemblies that are proposed to participate in cognitive processes.

## Author Summary

We recorded the first connections, to our knowledge, between human nerve cells and reveal that a subset of interactions is so strong that some presynaptic cells are capable of eliciting action potentials in the postsynaptic target neurons. Interestingly, these strong connections selectively link pyramidal cells using the neurotransmitter glutamate to neurons releasing gamma aminobutyric acid (GABA). Moreover, the GABAergic neurons receiving the strong connections include different types: basket cells, which inhibit several target cell populations, and another type called the chandelier cells, which can be excitatory and target pyramidal cells only. Thus, the activation originating from a single pyramidal cell propagates to synchronously working inhibitory and excitatory GABAergic neurons. Inhibition then arrives to various neuron classes, but excitation finds only pyramidal cells, which in turn, can propagate excitation even further in the network of neurons. This chain of events revealed here leads to network activation approximately an order of magnitude longer than detected previously in response to a single action potential in a single neuron. Individual-neuron–activated groups of neurons resemble the so-called functional assemblies that were proposed as building blocks of higher order cognitive representations.

## Abstract 3

A novel study on connections between human neurons reveals that single spikes in pyramidal cells can activate synchronously timed assemblies through strong connections linking pyramidal cells with inhibitory and excitatory GABAergic neurons.

## Introduction

Functional characterization of microcircuits of the cerebral cortex of rodents, carnivores, and to some extent, monkeys has been propelled by simultaneous multiple recordings from synaptically connected neurons combined with anatomical and molecular analysis of the recorded cells providing direct experimental analysis of neural connections [1–5]. In the human cortical microcircuit, however, only single cells were characterized to date; interactions between identified neurons were not studied [6–8].

Recent in vivo experiments in rodents showed that individual neurons of the cerebral cortex can effectively initiate movements [9] and modulate behavioral tasks [10]. This suggests that the activity of a single cell is sufficient for driving a relatively widespread functional assembly of neurons. However, mechanisms at the level of microcircuits are not clear in producing single-neuron–triggered events requiring the activation of neural assemblies originally postulated to be important in higher order brain functions by Hebb [11]. Feed-forward excitatory and inhibitory connections are required for wiring spike and signal propagation in neural circuits and were proposed to participate in sculpting the pattern of firing traveling through the network [11–20]. Experiments testing the effectiveness of individual pyramidal neurons of the cortex showed excitatory, but usually subthreshold, postsynaptic potentials and occasional triggering of postsynaptic spikes in interneurons, leading to temporally limited (<3 ms) microcircuit events terminated by disynaptic inhibitory responses [2,18,21–23]. Thus, it is considered that single presynaptic spikes in pyramidal cells are not sufficient for initiating postsynaptic firing in glutamatergic neurons [24], and effective triggering of subsequent multistep event sequences characteristic to functional neuronal assemblies requires concomitant activation of several convergent inputs or repeated firing of single presynaptic cells.

We set out to record the first interactions between identified human pyramidal cells and their postsynaptic target neurons in order to characterize the saliency of single cells and the contribution of unitary signals to the triggering of compound network events. Our recordings reveal that single spikes of pyramidal neurons are followed, not only by monosynaptic excitatory postsynaptic potentials (EPSPs), but also by complex event sequences with a stereotyped series of polysynaptic potentials. We then show some of the underlying network mechanisms that sequentially combine the pathway-specific effectiveness of glutamatergic excitation followed by a concomitant and bimodal GABAergic wave of events composed of inhibitory and excitatory effects.

## Results

**Figure 1:**
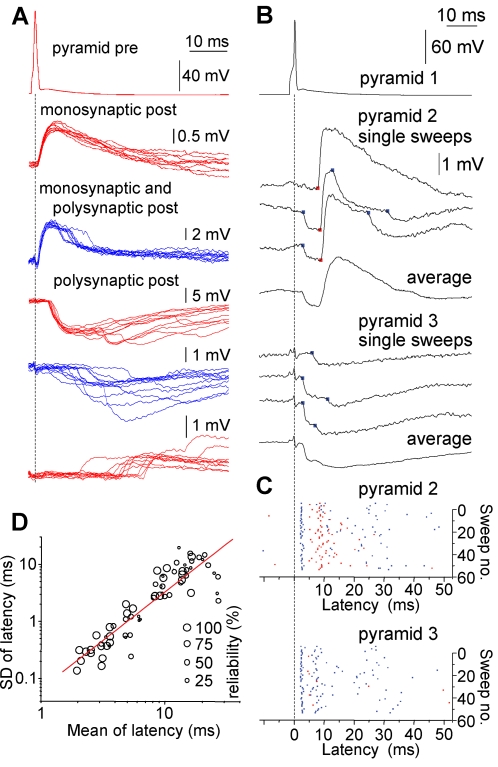
Polysynaptic Events in Postsynaptic Cells Initiated by Single Spikes of Human Pyramidal Cells (A) Individual presynaptic (Pre) action potentials in human pyramidal cells evoke mono- and polysynaptic responses in postsynaptic (Post) pyramidal cells (red) and interneurons (blue). Ten traces showing monosynaptic EPSPs (top), monosynaptic EPSPs followed by polysynaptic IPSPs (middle), and polysynaptic IPSPs and EPSPs (bottom) with increasing onset latencies. Polysynaptic events occurred in 100% of trials in each of these four experiments. The timing of the presynaptic action potential is indicated by the dashed line. (B) Single action potentials in pyramid 1–initiated sequences of multiple events in simultaneously recorded pyramidal cells (pyramids 2 and 3). Disynaptic IPSPs occurred synchronously on the two postsynaptic cells. Disynaptic IPSPs were followed by presumably polysynaptic EPSPs and IPSPs in pyramid 2 and downstream IPSPs in pyramid 3. Blue and red dots indicate polysynaptic IPSP and EPSP onset times, respectively. (C) Scatter diagram of 54 consecutive sweeps in response to single presynaptic spikes. Blue and red dots correspond to the onset of individual IPSPs and EPSPs, respectively. (D) Double logarithmic correlation between the standard deviation (SD) and the mean of latency of polysynaptic postsynaptic potentials.

We prepared brain slices from small blocks of nonpathological samples that had to be removed for the surgical treatment of brain areas beneath temporal (*n* = 30), frontal (*n* = 22), and parietal (*n* = 6) cortices of 58 nonepileptic patients aged 18 to 73 y [25]. Applying simultaneous dual, triple, and quadruple whole-cell recordings from cell groups (*n* = 429) in layer 2/3 of slices (*n* = 383), we elicited presynaptic action potentials in 681 pyramidal cells and searched for mono- and polysynaptic potentials in possible target neurons of which 252 were pyramidal cells and 481 were interneurons, reflecting a search bias towards interneurons during the experiments. As expected, single action potentials of pyramidal cells (*n* = 121) triggered monosynaptic EPSPs in simultaneously recorded pyramidal cells (*n* = 38) and interneurons (*n* = 93) with latencies of 0.91 ± 0.46 ms and 0.86 ± 0.32 ms (Figure 1A). Interestingly, single action potentials of pyramidal cells (*n* = 177) also triggered polysynaptic postsynaptic potentials in pyramidal cells (*n* = 144; latency, 10.02 ± 6.83 ms) and in interneurons (*n* = 82; latency 8.54 ± 6.19 ms) with latencies significantly longer than that of monosynaptic events (*p* < 0.001 for both; Figure 1A–1C). Postsynaptic pyramidal cells and interneurons were targeted by polysynaptic events with inhibitory (*n* = 195 and *n* = 96, respectively) and excitatory (*n* = 12 and *n* = 5, respectively) polarity, and several polysynaptic events could arrive at the same postsynaptic pyramidal cell (*n* = 22) or interneuron (*n* = 13; Figure 1A–1C). The frequency of single-spike–initiated synaptic potentials was increased for 37 ± 17 ms (range, 5–65 ms) compared to the control period that was measured 0–10 ms prior to the action potential (Figure 1A–1C). Thus, single spikes of individual pyramidal cells initiated a series of synaptic potentials with latencies approximately ten times longer than detected previously [2,18,21–23]. The standard deviation of the latency of polysynaptic connections showed a double logarithmic correlation with the latency (*R* = 0.90, *p* < 0.001, Figure 1D), but the probability of polysynaptic events was not correlated with their latency. Polysynaptic events in response to single spikes were detected in 105 out of 383 slices (27%) of samples taken from 54 out of 58 (93%) patients, showing no correlation with the cortical area from which slices were cut or with the age, diagnosis, and medication of the patients. This suggests that detected polysynaptic events are based on properties intrinsic to the human microcircuit that are conserved across brain areas.

**Figure 2:**
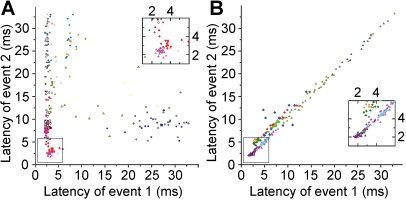
Relative Timing of Pairs of Polysynaptic Postsynaptic Potentials Detected in One or Several Postsynaptic Neurons within an Event Series Triggered by a Single Spike of Human Pyramidal Cells (A) Temporally uncorrelated event sequences triggered by single presynaptic spikes: the latency of event 1 does not predict the timing of a different polysynaptic event (event 2). (B) Groups of postsynaptic potentials with temporally correlated event sequences: the latency of event 1 shifts together with that of event 2 relative to the trigger spike. Correlated latencies of pairs of polysynaptic potentials occur up to 35 ms after the single action potential initiating the series of network events. Colors correspond to different experiments. Insets, magnification of the boxed region near zero on the graph.

Mono- and polysynaptic potentials in the network followed the trigger spike in a sequence of waves as observed on scattergrams representing the temporal pattern of events following unitary activation (Figure 1C). We searched for temporal correlations between synaptic events in single-neuron–activated networks, i.e., whether the latency of a polysynaptic postsynaptic potential would move together with that of a different event detected in the same or in a distinct cell relative to the trigger spike. Some events showed no significant correlation with preceding or following synaptic responses, presumably due to the aforementioned increase in the jitter of onset accompanying longer latencies (Figure 2A). However, we also found highly correlated (*R* > 0.84, *p* < 0.05) pairs of synaptic events composed of polysynaptic inhibitory postsynaptic potential (IPSP)—polysynaptic IPSP (*n* = 7) and polysynaptic IPSP—polysynaptic EPSP (*n* = 7) sequences that occurred up to 35 ms after the action potential (Figure 2B). Accordingly, the latency of some polysynaptic potentials moves together relative to the trigger spike and could be temporally referenced, not only to the initial action potential, but also to intermediate synaptic events, indicating firing of other neurons. This suggests that a single unitary signal is sufficient for activating a predefined sequence of action potentials traveling through a subset of neurons that do not necessarily receive direct input from the trigger cell and thus correspond to cell assemblies postulated by Hebb [11].

**Figure 3:**
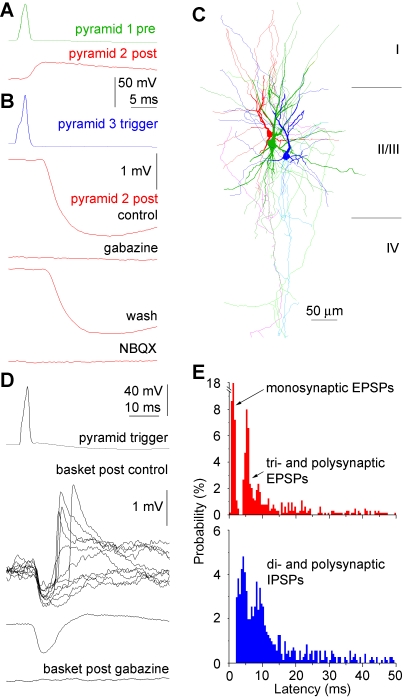
Sequential Polarity and Pharmacology of Single Pyramidal Spike–Triggered Polysynaptic Events in the Human Microcircuit (A–C) Polysynaptic IPSPs are mediated by GABAA receptors and require the activation of AMPA-type glutamate receptors. (A) Monosynaptic connection between pyramidal cells 1 and 2. (B) A third pyramidal cell (pyramid 3) triggers an inhibitory response in pyramid 2, but the response has a longer latency than the monosynaptic connections shown in (A). The inhibitory response is sensitive to both gabazine and NBQX, showing the involvement of GABAA and AMPA receptors. (C) Light microscopic reconstruction of the three pyramidal cells (colors correspond to the panels [A and B]). Post, postsynaptic; Pre, presynaptic. (D and E) Polysynaptic EPSPs require preceding activation of GABAA receptor mediated event(s) in the network. (D) Single spikes in a presynaptic pyramidal cell (top) trigger polysynaptic IPSPs and EPSPs in a postsynaptic fast-spiking basket cell (middle). The polysynaptic IPSPs as well as polysynaptic EPSPs are sensitive to the GABAA receptor antagonist gabazine (bottom). (E) Temporal distribution of EPSPs (top) and IPSPs (bottom) recorded in experiments when single spikes triggered polysynaptic EPSPs. Monosynaptic EPSPs were followed exclusively by disynaptic IPSPs, and thus polysynaptic EPSPs had minimally trisynaptic latencies. Tri- and polysynaptic EPSPs and all IPSPs were gabazine sensitive as shown in panel (D).

We applied pharmacological tools in search of mechanisms underlying unitary-spike–initiated network events. Application of the GABAA receptor antagonist gabazine (10 μM, *n* = 5) or the AMPA receptor antagonist NBQX (10 μM, *n* = 4) alone or consecutively (*n* = 6) effectively blocked pyramidal cell–triggered polysynaptic IPSPs (Figure 3A–3C). The dual sensitivity to glutamatergic and GABAergic antagonists suggests effective spike propagation from pyramidal cells to postsynaptic GABAergic neurons producing disynaptic IPSPs as shown earlier [2,18,21–23]. To date, however, pyramidal neurons were found to trigger disynaptic IPSPs only; further polysynaptic events in general, and EPSPs in particular, could not be initiated by individual pyramidal neurons. We found that human pyramidal neurons triggered depolarizing, polysynaptic EPSPs (*n* = 17) with latencies (11.88 ± 8.66 ms) distinct from that of monosynaptic EPSPs (Figure 3D and 3E, *p* < 0.001). Surprisingly, polysynaptic excitatory events were blocked by gabazine (*n* = 6) similar to polysynaptic inhibitory events (Figure 3D), indicating that a GABAergic step was required before the activation of downstream pyramidal neurons eliciting the polysynaptic excitation. Moreover, the temporal distribution of synaptic events recorded in experiments with single-spike–triggered polysynaptic EPSPs showed that monosynaptic EPSPs were followed exclusively by disynaptic IPSPs, and thus polysynaptic EPSPs had minimally trisynaptic latencies (Figure 3E). These experiments suggest that human pyramidal cells initially recruit postsynaptic spikes only in GABAergic neurons, then network mechanisms produce downstream IPSPs and EPSPs.

**Figure 4:**
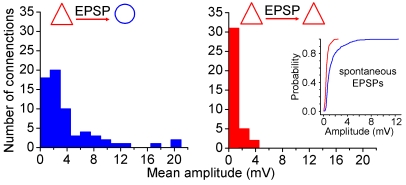
Amplitude Distribution of Unitary EPSPs Arriving at Pyramidal Cells (Bottom) and Fast-Spiking Interneurons (Top) from Local Pyramidal Cells in Layers 2/3 of the Human Cortex Note the unitary EPSPs of enormous amplitude selectively targeting fast-spiking interneurons. Inset, amplitude distribution of spontaneous EPSPs arriving at presynaptic human pyramidal cells (red) and postsynaptic interneurons (blue). The distribution of spontaneous EPSPs was shifted towards significantly higher amplitudes in interneurons relative to pyramidal cells.

**Figure 5:**
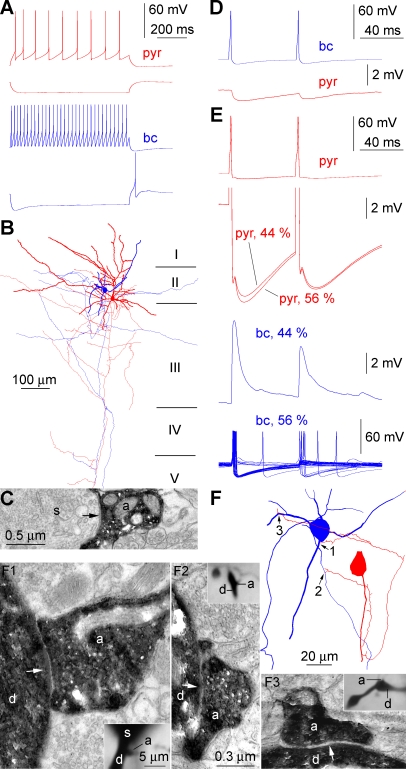
Spike-to-Spike Transmission from a Pyramidal Cell to a Basket Cell in the Human Neocortex (A) Firing patterns of the pyramidal cell (pyr; red) and the basket cell (bc; blue) and their responses to hyperpolarizing current pulses. (B) Light microscopic reconstruction of the pyramidal cell (red) and the basket cell (blue). (C) Electron micrograph showing an axon terminal of the basket cell forming a synaptic junction (arrow) on the soma (s) of an unlabeled neuron. (D) Action potentials in the basket cell (top) elicit monosynaptic IPSPs in the pyramidal cell (bottom). (E) Action potentials in the pyramid (red traces) evoke EPSPs (44% of trials) and EPSPs eliciting action potentials (56% of trials) in the basket cell (blue traces). When postsynaptic spikes are triggered, IPSPs arriving back from the basket cell increase the amplitude of the afterhyperpolarization following the action potential of the pyramidal cell. (F) Route of the presynaptic pyramidal axon to the synapses formed on the dendrites of the basket cell. (F1–F3) Correlated light and electron microscopic identification of the three synaptic junctions (arrows on electron micrographs) between the axon (a) of the pyramidal cell and dendrites (d) of the basket cell. Numbering of synapses correspond to panel (F).

**Figure 6:**
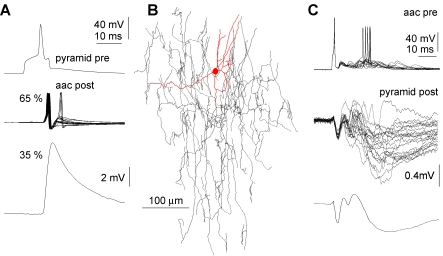
Human Axo-Axonic Cells Are Involved in Single Pyramidal Cell–Initiated Network Events (A) Single action potentials in a presynaptic (Pre) pyramidal neuron (top) were effective in triggering postsynaptic (Post) action potentials with high-amplitude unitary responses in a postsynaptic axo-axonic cell (aac). The action potentials in the pyramidal neuron resulted in monosynaptic EPSPs in 65% of the trials eliciting second-order action potentials (middle), whereas in 35% of the trials, only subthreshold EPSPs were evoked (bottom). Note that postsynaptic, second-order action potentials in the axo-axonic cell were followed by occasional higher order spikes riding on presumably trisynaptic EPSPs. (B) Reconstruction of the somatodendritic (red) and axonal (black) arborization of the postsynaptic axo-axonic cell shown in panel (A). (C) Action potentials in a human axo-axonic cell trigger a sequence of polysynaptic events following a monosynaptic IPSP and a disynaptic EPSP in a postsynaptic pyramidal cell recorded with a low intracellular chloride concentration rendering all IPSPs hyperpolarizing in the two recorded cells. Note that downstream recurrent activity in the network triggers spikes in the axo-axonic cell. Responses of the pyramidal cell are shown as single sweeps (middle) and the average (bottom).

As an initial test of this hypothesis, we performed simultaneous paired recordings and measured the amplitude of unitary EPSPs elicited by local pyramidal cells targeting pyramidal cells (*n* = 38) and fast-spiking interneurons (*n* = 65) recorded at resting membrane potentials of −72 ± 3 and −62 ± 4 mV, respectively (Figure 4). The firing behavior alone does not define a type of interneuron, thus we classified fast-spiking interneurons as basket (*n* = 56) and axo-axonic cells (*n* = 9), based on light microscopically identified axonal branches forming perisomatic baskets or characteristic axonal cartridges or candles, respectively (Figures 5B and 6B) [25–27]. According to similar intrinsic electrophysiological properties and EPSP characteristics, we pooled the data from basket and axo-axonic cells. The amplitude distribution of unitary glutamatergic inputs targeting pyramidal cells and fast-spiking interneurons differed significantly due to unitary EPSPs of high amplitude found in interneurons (*p* < 0.001, Kolmogorov-Smirnov test, Figure 4). Similar to what was found in other species [2,5,21,23,28], the input resistance of postsynaptic fast-spiking interneurons (134 ± 64 MΩ) was higher (*p* < 0.042) than that of pyramidal cells (102 ± 51 MΩ) receiving unitary EPSPs. However, the approximately 30% difference in input resistances could not account for the finding of unitary EPSPs with amplitudes bigger than approximately 6 mV in fast-spiking cells, and, furthermore, there was no correlation between the amplitude of unitary EPSPs and postsynaptic input resistance in pyramidal cells (*p* < 0.209; *R* ^2^ = 0.025) and in interneurons (*p* < 0.552; *R* ^2^ = 0.017). This suggests that a subset of connections targeting fast-spiking interneurons is selectively strengthened in the human cortical circuit. We also compared the amplitude distribution of spontaneous EPSPs arriving at the presynaptic pyramidal cells and postsynaptic fast-spiking cells (Figure 4). The distribution of spontaneous EPSPs normalized to input resistances was shifted towards higher amplitudes in interneurons relative to pyramidal cells (*p* < 0.001, Kolmogorov-Smirnov test), corroborating our data on unitary EPSPs. In line with our hypothesis, spike transmission between pyramidal cells was not detected, but unitary EPSPs could mediate direct spike-to-spike coupling (*n* = 14) with a probability of 46 ± 27% in individual connections from pyramidal cells to fast spiking interneurons held at resting membrane potential of −59 ± 3 mV (Figures 3D and 4A). The latency of postsynaptic action potentials in fast-spiking cells (3.34 ± 1.76 ms) was in the range of the latency of pyramidal cell–triggered disynaptic IPSPs. Subsequent light microscopic reconstruction of the connected cells and electron microscopic analysis of all potential sites of interaction in two of such connections showed that a relatively small number of ultrastructurally identified synaptic junctions (*n* = 3 and 6) scattered over the dendritic surface 29 ± 19 μm and 94 ± 34 μm from the soma of the postsynaptic basket cells was sufficient for mediating the spike-to-spike coupling (Figures 3F and S1).

**Figure 7:**
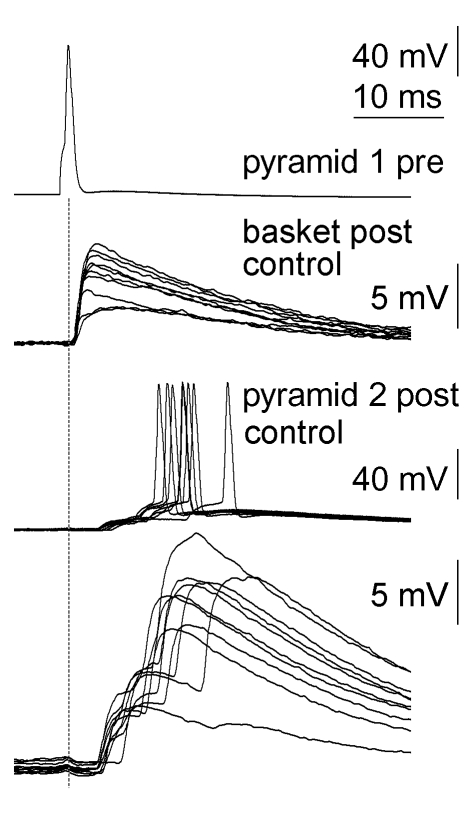
Polysynaptic Spike-to-Spike Coupling between Human Pyramidal Cells Unitary action potentials in pyramidal cell 1 (top) elicited subthreshold, monosynaptic EPSPs in the postsynaptic (Post) basket cell (middle) and late-onset action potentials riding on polysynaptic EPSPs in pyramidal cell 2 (bottom). The peak of the presynaptic (Pre) action potential is projected by a dashed line for easier comparison of mono- and polysynaptic EPSP latencies.

Single pyramidal spike–driven suprathreshold responses in inhibitory interneurons explain the occurrence of disynaptic IPSPs following action potentials of pyramidal cells [2,18,21–23] but do not provide the mechanism for the recruitment of gabazine-sensitive polysynaptic excitatory events at trisynaptic latencies. However, postsynaptic action potentials were triggered, not only in 20% of GABAergic basket cells known to elicit hyperpolarizing IPSPs (*n* = 11, Figure 5), but also in 33% of axo-axonic or chandelier cells (*n* = 3, Figure 6) capable of triggering depolarizing IPSPs and postsynaptic action potentials in neocortical pyramidal cells [25,29]. Axo-axonic cells were shown to be effective in the selective recruitment of postsynaptic glutamatergic neurons and disynaptic EPSPs in the cortex and amygdala [25,30]. Thus, spike transmission from pyramidal to axo-axonic cells and then from axo-axonic cells to pyramidal neurons could form the mechanism underlying single pyramidal cell–triggered, trisynaptic, and gabazine-sensitive EPSPs suggested also by experiments detecting single action potential–initiated polysynaptic recruitment of downstream spikes (*n* = 4, Figure 7). Furthermore, all human axo-axonic cells in our sample (*n* = 9) triggered polysynaptic series of events with latencies corresponding to monosynaptic GABAergic, disynaptic glutamatergic, and/or trisynaptic GABAergic postsynaptic potentials (Figure 6A and 6C). The resulting alternating sequence of inhibitory and excitatory waves of responses were similar to brief, high-frequency oscillations (175 ± 28 Hz, measured between the peaks of inhibitory components taken as troughs, Figure 6C).

## Discussion

Our results show that a single spike of a pyramidal cell in the human cortical microcircuit is capable of activating complex sequences of postsynaptic potentials lasting an order of magnitude longer than detected previously [2,18,21–23]. The initiation and internally precise temporal structure of these event series appears to follow a stereotyped mechanism of spike-to-spike transmission traveling through a subset of synaptically connected neurons that seems to be conserved across several brain regions of the human cerebral cortex. The flow of downstream activation that follows the first-order spike of the trigger pyramidal cell is directionally controlled at two consecutive synaptic steps. Second-order spikes are triggered exclusively in GABAergic interneurons and not in pyramidal cells due to interneuron-selective EPSPs of enormous amplitude. In turn, second-order spikes in axo-axonic cells give rise to third-order spikes detected only in pyramidal cells, resulting in trisynaptic EPSPs in the network because axo-axonic cells do not innervate other GABAergic cells [26,27]. Synchronized to the spikes in axo-axonic cells, second-order spikes in basket cells and possibly in other types of interneuron [18,23] elicit hyperpolarizing effects reported here as disynaptic IPSPs. Conversely, the IPSPs hyperpolarizing pyramidal cells could enhance the excitatory effect of axo-axonic cells by pushing the postsynaptic membrane potential further from the relatively depolarized axonal reversal potential for GABA [25,29], especially during the second spike of spike doublets recorded frequently in cortical and hippocampal axo-axonic cells [25,31]. Moreover, hyperpolarizing IPSPs suppress the activity of pyramidal cells not recruited by axo-axonic cells, decreasing the frequency of EPSPs not routed to the initial spike. Thus, the actual pattern of single-cell–initiated network activity, especially during the first two to three synapses, could be shaped by the convergence and divergence relationship of inhibitory and excitatory GABAergic synapses in addition to glutamatergic recruitment. The clockwork precision of these mono-, di-, and trisynaptic events of alternating glutamatergic and GABAergic transmission resembles sharp, wave-like oscillation hotspots in the microcircuit [27,32–35]. At later stages of the polysynaptic chain, these mechanisms are likely to be combined with summation of glutamatergic EPSPs leading to suprathreshold responses in pyramidal cells in addition to interneurons and with rebound excitation following IPSPs at a relatively longer delay [16].

Synapses arriving from a single human pyramidal cell are not capable of driving the postsynaptic pyramidal cells to fire. Accordingly, there are two alternatives for eliciting suprathreshold responses in a pyramidal cell from local sources: (1) single axo-axonic cell-to-pyramid connections [25] and (2) synchronous inputs from several pyramidal/glutamatergic cells converging onto the same postsynaptic pyramidal cell [24]. Importantly, axo-axonic cells recruit the firing of several postsynaptic pyramidal cells in synchrony [25], and thus they can contribute to pyramid–pyramid spike-to-spike coupling (Figure 7). The finding that polysynaptic EPSPs are relatively rarely found could be due to the limited number of axo-axonic cells relative to basket cells or to the limited percentage of pyramidal cells responding with a spike to axo-axonic input [25]. This, however, could be crucial in preventing overexcitation of the microcircuit and can effectively dampen the detonator effect of axo-axonic cells. Our data suggest that axo-axonic cells are crucial in the distribution of local excitation originating from sporadic spikes in pyramidal cells, but pyramid–pyramid connections are highly convergent in the cortex [36], thus sufficient synchronization of inputs could occur during various cortical operations.

The dataset presented here does not provide sufficient evidence to decide whether single-EPSP–triggered, long-lasting event series are specific to the human cerebral cortex. Interestingly, previous studies testing the effect of identified pyramidal cells in the monkey cortex did not report recruitment of polysynaptic activity [5]. When searching for similar, single pyramidal cell–triggered polysynaptic postsynaptic potentials in our library of recordings performed in the somatosensory and prefrontal cortex of the rat (*n* > 4,500 unitary connections), we could only detect sporadic triggering of polysynaptic events that were IPSPs only (*n* = 8) apart from the unitary EPSPs (*n* = 724) elicited by layer 2/3 pyramidal neurons. Although the ratio of triggering poly- versus monosynaptic postsynaptic potentials was 0.01 in the rat and 1.73 in the human in our hands, it should be emphasized that the human patients were treated differently during anesthesia and surgery, and the excitability of human neurons might be different in the external solution also used for rat experiments. Applying similar constraints, unitary EPSPs of high amplitude arriving to human interneurons were not reported in other species to date. It is apparent that the potential amplitude difference in unitary EPSPs between human and nonhuman interneurons is not attributable to interspecies differences in input resistances [2,5,21,23,28]. Quantal parameters of synaptic transmission underlying gigantic EPSPs on subsets of human GABAergic interneurons are not known and are being tested as part of a separate study.

The function of single-spike–initiated event series is not clear, although they fit into the framework of the cell assembly concept proposed by Hebb [11]. The present study identifies that Hebbian-like cell assemblies can be recruited by a unitary signal of a single pyramidal cell and suggests a canonical order of activation of identified cell populations assigning the numbers representing the sequence of active connections in the original illustration of Hebb [11]. Our data support the idea of specialized and strong pathways in the cortex among the many weaker pathways [11,20,33,35,37] and identifies a novel cell population, the axo-axonic cells, selectively targeted by strong inputs from individual pyramidal neurons. Mechanisms producing the powerful glutamatergic synapses on axo-axonic and basket cells are not fully understood. Interestingly, it has been suggested that a special form of LTP may occur in interneurons that are silent during periods of intense pyramidal cell firing, such as sharp waves [38], and axo-axonic cells fire in vivo before, but not during sharp waves [34]. Feed-forward inhibition provides a framework for efficient propagation and selection of excitatory events [18,22] especially when synchronized to the feed-forward GABAergic excitation and recruitment of a subset of glutamatergic neurons leading to the correlated action of neurons several synapses downstream in the activated network. The increased signal-to-noise ratio in the network provided by hyperpolarizing GABAergic synapses is further amplified by the coincident action of chandelier cells, resulting in a sparse and potentially task-selective activation of pyramidal neurons. Thus, the human microcircuit appears to be tuned for unitary-EPSP–activated Hebbian-like functional cell assemblies [11,37,39] that were proposed as building blocks of higher-order cortical operations and could contribute to single cortical cell–initiated movements [9] and behavioral responses [10].

## Materials and Methods

### Electrophysiology and analysis.

All procedures were performed according to the Declaration of Helsinki with the approval of the University of Szeged Ethical Committee. Human slices were derived from material that had to be removed to gain access for the surgical treatment of deep-brain tumors from the left and right frontal, temporal, and parietal regions with written informed consent of the patients (aged 18–73 y) prior to surgery over the last 4 y. Anesthesia was induced with intravenous midazolam and fentanyl (0.03 mg/kg, 1–2 μg/kg, respectively). A bolus dose of propofol (1–2 mg/kg) was administered intravenously. To facilitate endotracheal intubation, the patient received 0.5 mg/kg rocuronium. After 120 s, the trachea was intubated and the patient was ventilated with a mixture of O_2_-N_2_O at a ratio of 1:2. Anesthesia was maintained with sevoflurane at monitored anesthesia care (MAC) volume of 1.2–1.5. Blocks of tissue were immersed in ice-cold solution containing (in mM) 130 NaCl, 3.5 KCl, 1 NaH_2_PO_4_, 24 NaHCO_3_, 1 CaCl_2_, 3 MgSO_4_, 10 d(+)-glucose, saturated with 95% O_2_ and 5% CO_2_ in the operating theatre, sliced at a thickness of 350 μm with a vibrating blade microtome (Microm HM 650 V) and were incubated at room temperature for 1 h in the same solution. The solution used during recordings differed only in that it contained 2 mM CaCl_2_ and 1.5 mM MgSO_4_. Recordings were obtained at approximately 36 °C from up to four concomitantly recorded cells visualized in layer 2/3 by infrared differential interference contrast videomicroscopy at depths 60–130 μm from the surface of the slice. Signals were filtered at 8 kHz, digitized at 16 kHz, and analyzed with PULSE software. Micropipettes (5–7 MΩ) were filled with a low [Cl]i solution for discriminating GABAergic and glutamatergic events containing (in mM) 126 K-gluconate, 4 KCl, 4 ATP-Mg, 0.3 GTP-NA_2_, 10 HEPES, 10 phosphocreatine, and 8 biocytin (pH 7.20; 300 mOsm). Presynaptic cells were stimulated with brief (2–10 ms) suprathreshold pulses delivered at >7-s intervals, to minimize intertrial variability. Membrane properties of human neurons or polysynaptic events did not show significant changes for up to 20 h after slicing, but recordings included in the analysis were arbitrarily terminated 15 h after slice preparation. Traces shown are single sweeps or averages of 50–100 consecutive episodes. Data are given as mean ± standard deviation (S.D.), Mann-Whitney *U*-test, paired *t*-test (pharmacology), and Kolmogorov-Smirnov test was used to compare datasets; differences were accepted as significant if *p* < 0.05.

### Histology.

Visualization of biocytin and correlated light and electron microscopy was performed as described [25,40]. Three-dimensional light microscopic reconstructions were carried out using Neurolucida with 100× objective.

## Supporting Information

Figure S1 Spike-to-Spike Transmission from a Pyramidal Cell to a Basket Cell in the Human Neocortex (A) Firing patterns of the pyramidal cell (pyr; red) and the basket cell (bc; blue) and their responses to hyperpolarizing current pulses.
(B) Action potentials in the pyramid (red) evoke subthreshold EPSPs (in 26% of trials) and EPSPs eliciting action potentials (in 74% of trials) in the basket cell (blue).
(C) Light microscopic reconstruction of the pyramidal cell (red) and the basket cell (blue).
(D) Route of the presynaptic pyramidal axon to the six electron microscopically verified synapses formed on the dendrites of the basket cell.
(E) Correlated light (insets) and electron microscopic (E1–E5) identification of the six synaptic junctions (arrows on electron micrographs) between the axon (a) of the pyramidal cell and dendrites (d) of the basket cell. Numbering of synapses correspond to panel (D).
(9.10 MB TIF)

## Abbreviations

EPSP: excitatory postsynaptic potential
IPSP: inhibitory postsynaptic potential
